# Revisiting meniscal anatomical variants of the knee: a high-resolution 7-Tesla MRI study

**DOI:** 10.1007/s00330-025-12090-2

**Published:** 2025-10-29

**Authors:** Roy P. Marcus, Adrian A. Marth, Benjamin Fritz, Stefan M. Zimmermann, Daniel Nanz, Reto Sutter

**Affiliations:** 1 Department of Radiology, Balgrist University Hospital Zurich, Zurich, Switzerland; 2 Faculty of Medicine, University of Zurich, Zurich, Switzerland; 3 Swiss Center for Musculoskeletal Imaging, Balgrist Campus AG, Zurich, Switzerland; 4 Department of Orthopedic Surgery, Balgrist University Hospital, Zurich, Switzerland

**Keywords:** Knee, 7-T magnetic resonance imaging, Meniscus, Roots, Anatomical variations

## Abstract

**Objectives:**

To examine anatomical variants of meniscal root insertions among asymptomatic individuals using high-resolution 7-T MRI.

**Materials and methods:**

This prospective study, approved by the local ethics committee, involved 57 knees from 48 participants (mean age 31.8 years; 19 females), being examined on a clinical 7-T MRI scanner with sagittal isotropic 0.24 mm 3D double echo steady state (DESS) sequence. Two radiologists identified the number and location of each meniscal root insertion. Two other radiologists repeated this evaluation, noting meniscofemoral ligaments. Inter-rater reliability was calculated.

**Results:**

Majority of the posterior lateral meniscus roots featured double insertions (66.7%), followed by single (31.6%) and triple insertions (1.8%). Single roots mainly inserted into the intercondylar area (77.8%), the remaining into the posterior slope of the posteromedial eminence (22.2%). Double roots typically had a major insertion at the posteromedial aspect of the anterior cruciate ligament and a minor root at the posterior slope of the lateral eminence (78.9%). The remaining roots of both menisci featured only single insertions. Anterior medial roots inserted along the medial tibial edge (71.9%) and into the intercondylar area (28.1%). Inter-rater reliability for describing the posterior lateral meniscal root insertions was perfect (κ = 1), and strong for identifying all insertion sites (κ = 0.808). Meniscofemoral ligaments were prevalent (87.7%) with a broad heterogenous fan-shaped femoral attachment observed using the 3D DESS sequence.

**Conclusion:**

7-T 3D-DESS imaging allows high-resolution visualization of the meniscal roots with excellent inter-rater reliability, highlighting anatomical variability mainly in the posterior root of the lateral meniscus.

**Key Points:**

***Question*** *Anatomical variations of the number and insertion sites of meniscal roots are documented in anatomical and imaging studies.*

***Findings*** *High-resolution 3D dual-echo steady state on a 7-T MRI facilitates the visualization of the number of roots and their respective osseous insertions for both menisci.*

***Clinical relevance*** *The menisci demonstrate considerable variability in both root number and their insertion locations, particularly the posterior lateral meniscus exhibits up to three root insertions. Understanding anatomical variants is crucial for the accurate interpretation and reporting of knee imaging.*

**Graphical Abstract:**

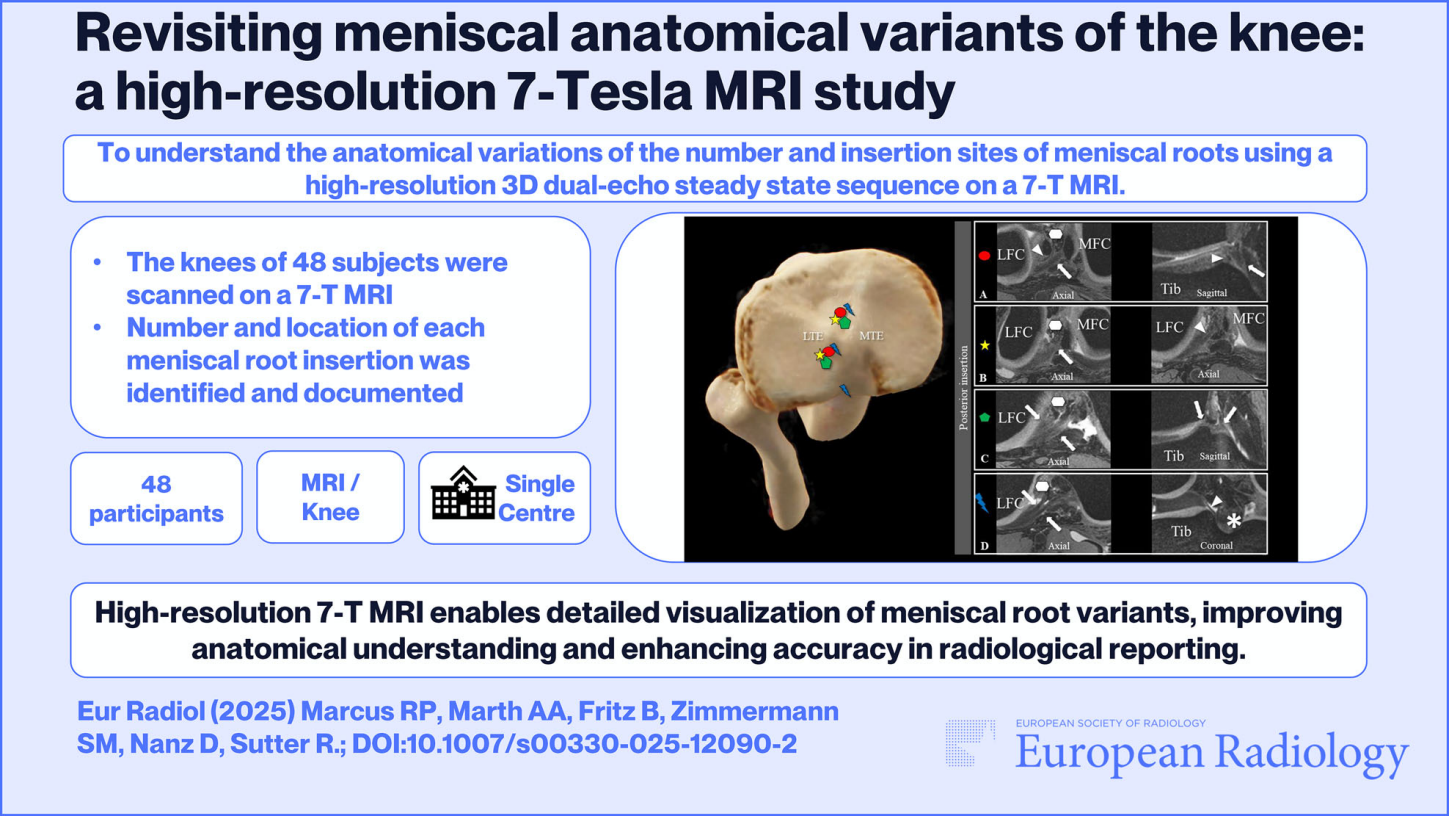

## Introduction

Menisci play a crucial role in knee biomechanics and have essential functions in knee kinematics, load distribution, and load transmission across the joint [1]. Meniscal roots are of particular relevance, as meniscal root tears profoundly alter the biomechanical characteristics of wedge-shaped menisci, and chronic meniscal root tears can accelerate the onset of osteoarthritis [2–5]. Arthroscopic assessment of the posterior compartment of the knee poses inherent challenges because of limited visibility with standard arthroscopy portals, and posterior meniscal root tears are often missed when relying solely on an anterior arthroscopic access [6, 7]. MRI of the knee plays an important role in detecting meniscal root lesions and can help guide surgeons to otherwise hidden meniscal root tears. Several anatomical variations of the menisci are known from cadaveric, arthroscopic, and MRI studies, with most variants found at the posterior root of the lateral meniscus [8–11]. The introduction of clinical 7-T MRI has advanced musculoskeletal imaging, offering improved spatial and temporal resolution compared to 3-T MRI [12–14]. These improvements have been particularly beneficial in studies focused on knee cartilage [15, 16]. With its increased contrast and higher signal-to-noise ratio (SNR), 7-T MRI can detect subtle morphological changes and enhance overall diagnostic confidence. However, it does not necessarily reveal previously undetected lesions [17, 18]. Despite these advantages, high-resolution 7-T MRI has yet to be extensively utilized to investigate meniscal anatomy.

This prospective study investigated anatomical variations regarding the number of meniscal root insertions and their corresponding insertion locations in asymptomatic subjects using 7-T MRI and employing a high-resolution 3D double-echo steady-state sequence (DESS).

## Materials and methods

This prospective study was approved by the local ethics committee. All participants had given written informed consent.

### Participants

**Fig. 1:**
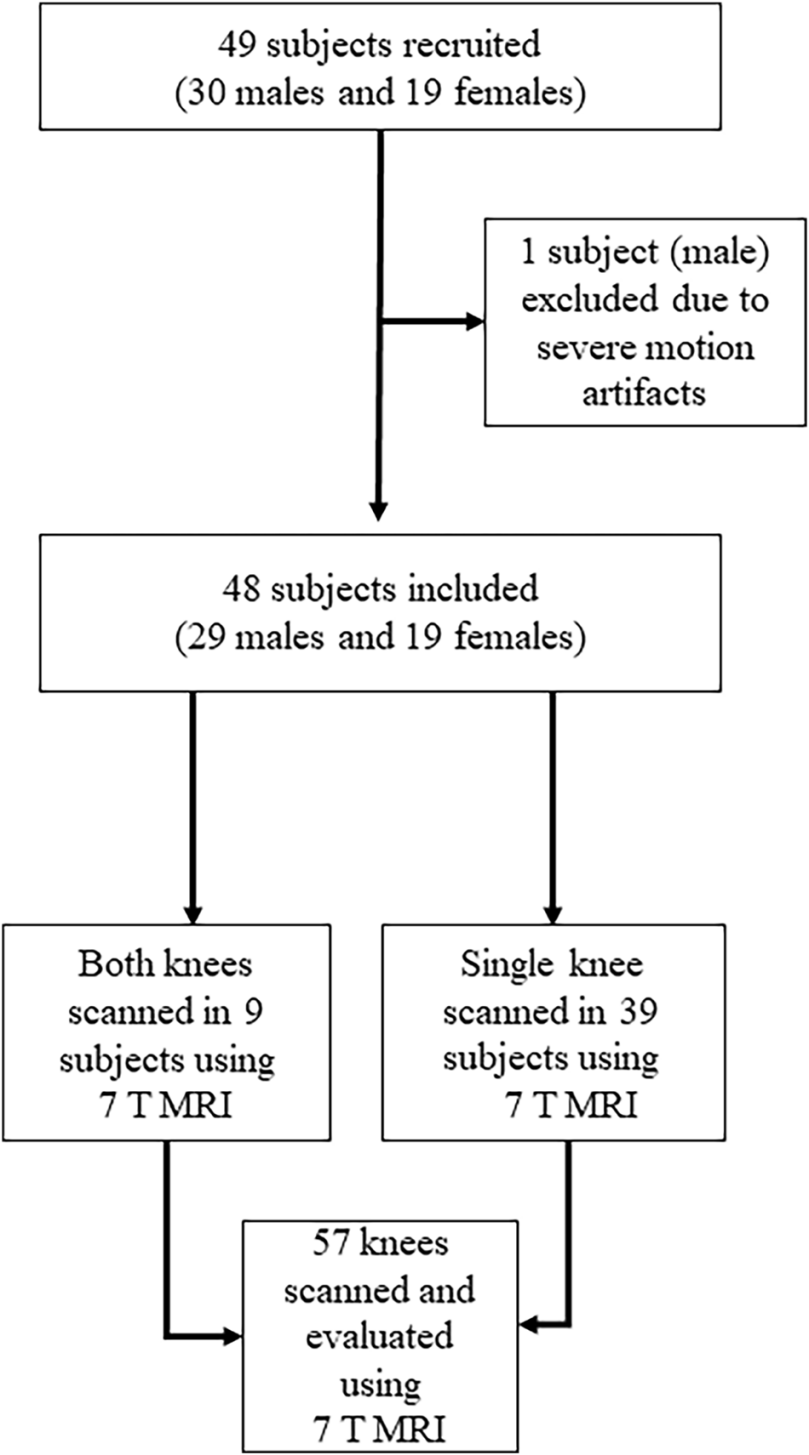
Flowchart illustrating the study design of healthy participants undergoing knee examination using 7-T MRI

Forty-nine participants were recruited between April 2021 and March 2023. The study included only asymptomatic individuals over 18 years of age who had no prior knee surgeries (Fig. 1; Table 1). Individuals with claustrophobia or metal implants, such as knee endoprostheses, were excluded.

**Table 1:** Demographics of the participants

Characteristics	Value
Number of subjects recruited	49
Number of subjects included	48
Subjects having one knee scanned	39
Subjects having both knees scanned	9
Total knees scanned and evaluated	57
Right knee scanned	32
Left knee scanned	25
Females	19
Males	29
Mean age in years ± standard deviation	32.2 ± 8.8

### Data acquisition

All subjects were scanned on a clinical 7-T MRI scanner (Magnetom Terra, Siemens Healthineers) with a dedicated 28-channel transmit/receive knee coil using an isotropic 3D sagittal DESS sequence with a field of view of 160 × 160 × 123 mm and a reconstructed voxel size of 0.20 × 0.20 × 0.24 mm^3^. Detailed scanning parameters are listed in Table 2.

**Table 2:** Scanning parameters applied for the dedicated knee acquisition on a 7-T MRI using a 28-channel transmit/receive knee coil

	3D Sagittal Water-excitation DESS
Field of view, mm^2^ (ro, phase, slice)^a^	160 × 160 × 123
Encoded matrix (ro, phase, slice)^a^	400 × 400 × 282
Reconstructed matrix (ro, phase, slice)^a^	800 × 800 × 512
Reconstructed voxel dimensions, mm^3^	0.20 × 0.20 × 0.24 = 0.0096
Inter-slice gap, mm	0.0
Phase-encoding direction	Head » Foot
Oversampling in phase- and slice directions, %^a^	62, 0.0
Receive bandwidth, Hz Pixel^−^^1^	695
(Refocusing) Flip angle, degrees	20
TR, TE, ms	8.5, 2.7
Parallel-imaging acceleration factor, phase (GRAPPA)^a^	3
Number of acquired reference lines	32
Partial Fourier in phase- and slice directions, fraction^a^	7/8, 7/8
Number of averages	1
Acquisition time, min:s	7:12

*DESS* double echo steady state, *TR* repetition time, *TE* echo time, *Hz* Hertz

^a^ ro: readout direction, phase: phase-encoding direction, slice: through-slice direction

### Structural evaluation

The menisci were structurally evaluated in three reading rounds on a clinical Picture Archiving and Communication System (PACS) workstation (Merlin Diagnostic Workstation v. 7.1, Phönix PACS). All images were anonymized, and readers were allowed to manually adjust the multiplanar reconstructions of the 3D dataset to best visualize the meniscal anatomy.

1) In the first round, two fellowship-trained musculoskeletal radiologists with > 10 years of clinical experience (R.P.M. and R.S.) evaluated the 3D datasets in a consensus fashion for meniscal tears and intact meniscal roots.

2) In the second round, the same two fellowship-trained musculoskeletal radiologists re-evaluated the 3D datasets of subjects with intact roots in consensus using multiplanar reconstruction (MPR). They identified the number of meniscal root insertions and mapped the anatomical locations of the tibial insertional areas. Additionally, both radiologists assessed the signal characteristics of the menisci and meniscofemoral ligaments.

3) In the third round, the same 3D datasets were independently reviewed by two fellowship-trained radiologists with > 10 years (B.F.) and 5 years of clinical experience (A.A.M.). They independently evaluated the number and anatomical locations of all meniscal root insertions using the map established in the first round. Additionally, they assessed the presence of the anterior (Humphrey) and posterior (Wrisberg) meniscofemoral ligaments, as well as the presence of a discoid meniscus.

4) In the fourth round, a fellowship-trained radiologist with > 10 years of clinical experience (R.P.M.) quantified the insertion areas of all four meniscal roots and their anatomic relationships to key arthroscopic anatomical landmarks using MPR, described by Johannsen et al and LaPrade et al [19, 20]. In addition, the extent of the meniscofemoral ligament attachment into the posterior aspect of the lateral meniscus was assessed using MPR. This was defined as the distance over which the slightly hyperintense meniscofemoral ligament, running parallel to the posterior horn of the lateral meniscus, remained distinguishable.

### Statistics

Samples were tested for normal distribution using the Shapiro-Wilk test. Normally distributed data are displayed as mean ± standard deviation, whereas non-normally distributed data are represented as median and range. Cohen’s kappa (κ) coefficient was calculated for assessing the inter-rater reliability [21]. Agreement values were interpreted as follows: κ = 0–0.2 (none), κ = 0.21–0.39 (minimal), κ = 0.4–0.59 (weak), κ = 0.6–0.79 (moderate), κ = 0.8–0.9 (strong) and κ > 0.9 (almost perfect) [22]. The significance level was set at *p* < 0.05.

Statistical analysis was performed using IBM SPSS Statistics for Windows version 28 (IBM Corp Released 2021).

## Results

Forty-nine participants were recruited for the study, and one participant had to be excluded due to severe motion artifacts. This resulted in a study cohort of 48 subjects (19 women and 29 men; mean age of 32.2 ± 8.8 years. Nine participants (19%) had both knees scanned. A total of 57 knees were evaluated (32 left and 25 right knees; Fig. 1). All scanned knees exhibited intact meniscal root insertions. Meniscal tears were observed in six knees from subjects aged 31, 35, 36, 51 and 55 years, primarily affecting the pars intermedia and posterior meniscal horn.

### Morphology

#### Menisci

**Fig. 2:**
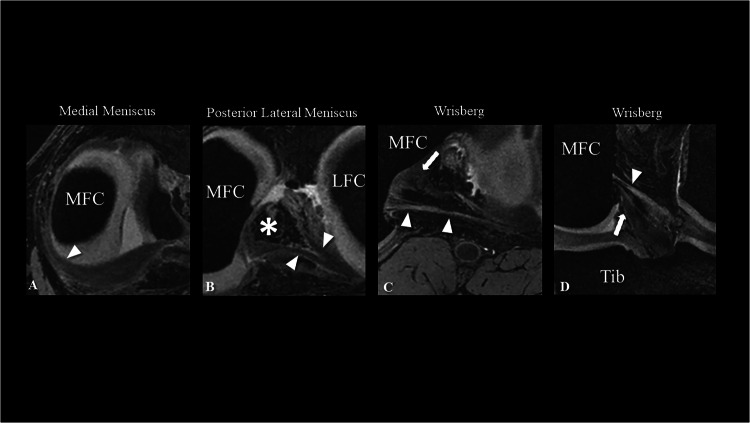
An exemplary image series showing the structural details of the left knee of a 40-year-old male participant using the 3D DESS sequence. **A** Morphology and signal of the medial meniscus in the axial plane; arrowheads highlight the fine striations. **B** Posterior lateral meniscus anatomy in the axial plane: arrowheads highlight the two inserting roots. The asterisk indicates the anterior cruciate ligament insertion. Morphology of the posterior meniscofemoral ligament (Wrisberg ligament) in the axial oblique (**C**) and coronal oblique planes (**D**): The arrowheads indicate the Wrisberg ligament, while the arrows indicate the PCL. The high spatial resolution enables a detailed visualization of the fan-shaped femoral insertion of the Wrisberg ligament. MFC, medial femoral condyle; LFC, lateral femoral condyle; PCL, posterior cruciate ligament; Tib, tibia

The signal intensity of the menisci was hypointense compared with that of the muscle in the 3D DESS sequence, exhibiting fine horizontal hyperintense striations (Fig. 2).

#### Meniscal roots

The meniscal roots seamlessly continued from the meniscus, displaying a hyperintense signal and structure compared to the homogeneous posterior cruciate ligament and hypointensity relative to the adjacent anterior cruciate ligament fibers.

#### Meniscofemoral ligaments

Both anterior and posterior meniscofemoral ligaments displayed a cylindrical morphology, iso-intense to the meniscus and hypointense to the adjacent cartilage. As for the femoral attachment, both the anterior and posterior meniscofemoral ligaments exhibited hyper- and hypointense striated fan-shaped structures.

### Insertion sites: menisci

Two insertion sites were identified for the anterior medial root: the medial tibial edge and the intercondylar area. Regarding the posterior medial root, the downslope of the posterior intercondylar fossa was determined to be the only insertion site. The anterior lateral root inserted at a single site in the intercondylar area, closely adjacent to and partially overlapping the tibial insertion of the anterior cruciate ligament (ACL).

In contrast, the posterior lateral root demonstrated four distinct insertion sites, located in close proximity to the insertion of the medial lateral root: posteromedial to the tibial ACL-insertion, the posterior slope of the lateral tibial eminence, the posterior slope of the medial eminence and posterior to the tibial insertion of the PCL (posterior cruciate ligament) (Table 3).

**Table 3:** Illustration of various insertion sites and the corresponding number of possible roots for each meniscus

Meniscus root	Number of possible roots	Pattern	Insertion sites	Insertion sites	Percentage	Root insertion area (mm^2^)	
						Total area	Individual areas
Anterior medial root	1	Type I	Medial tibial edge	3A	71.9 (41/57)	46.3 ± 9.1	
		Type II	Intercondylar area	3B	28.1 (16/57)	42.7 ± 11.3	
Posterior medial root	1	Type I	Downslope of the posterior intercondylar fossa	3C	100 (57/57)	34.5 ± 6.8	
Anterior lateral root	1	Type I	Intercondylar fossa adjacent to and partially overlapping the ACL	4A	100 (57)	50.2 ± 9.6	
Posterior lateral root	1	Type I	Intercondylar area	4B	77.8 (14/18)	33.8 ± 4.5	
		Type II	Posterior slope of the medial tibial eminence	4C	22.2 (4/18)	34.3 ± 7.8	
	2	Type I	Posterior medial to the tibial ACL insertion (major root)	5A	78.9 (30/38)	44.7 ± 8.8	32.3 ± 8.6
			Posterior slope of the lateral tibial eminence (minor root)				12.4 ± 3.2
		Type II	Posterior medial to the tibial ACL insertion (minor root)	5B	5.3 (2/38)	41.3 ± 11.2	34.6 ± 10.5
			Posterior slope of the lateral tibial eminence (major root)				6.7 ± 0.7
		Type III	Posterior slope of the medial tibial eminence	5C	15.8 (6/38)	39.3 ± 7.3	22.2 ± 6.3
			Posterior slope of the lateral tibial eminence				17.2 ± 4.9
	3	Type I	Posterior slope of the medial tibial eminence	5D	100 (1/1)	61.1	34.8
			Posterior slope of the lateral tibial eminence				23.5
			Posterior to the tibial insertion of the PCL				2.8

The number of roots ranged from one to three across the different insertion sites

*ACL* anterior cruciate ligament, *PCL* posterior cruciate ligament

### Medial meniscal variations

**Fig. 3:**
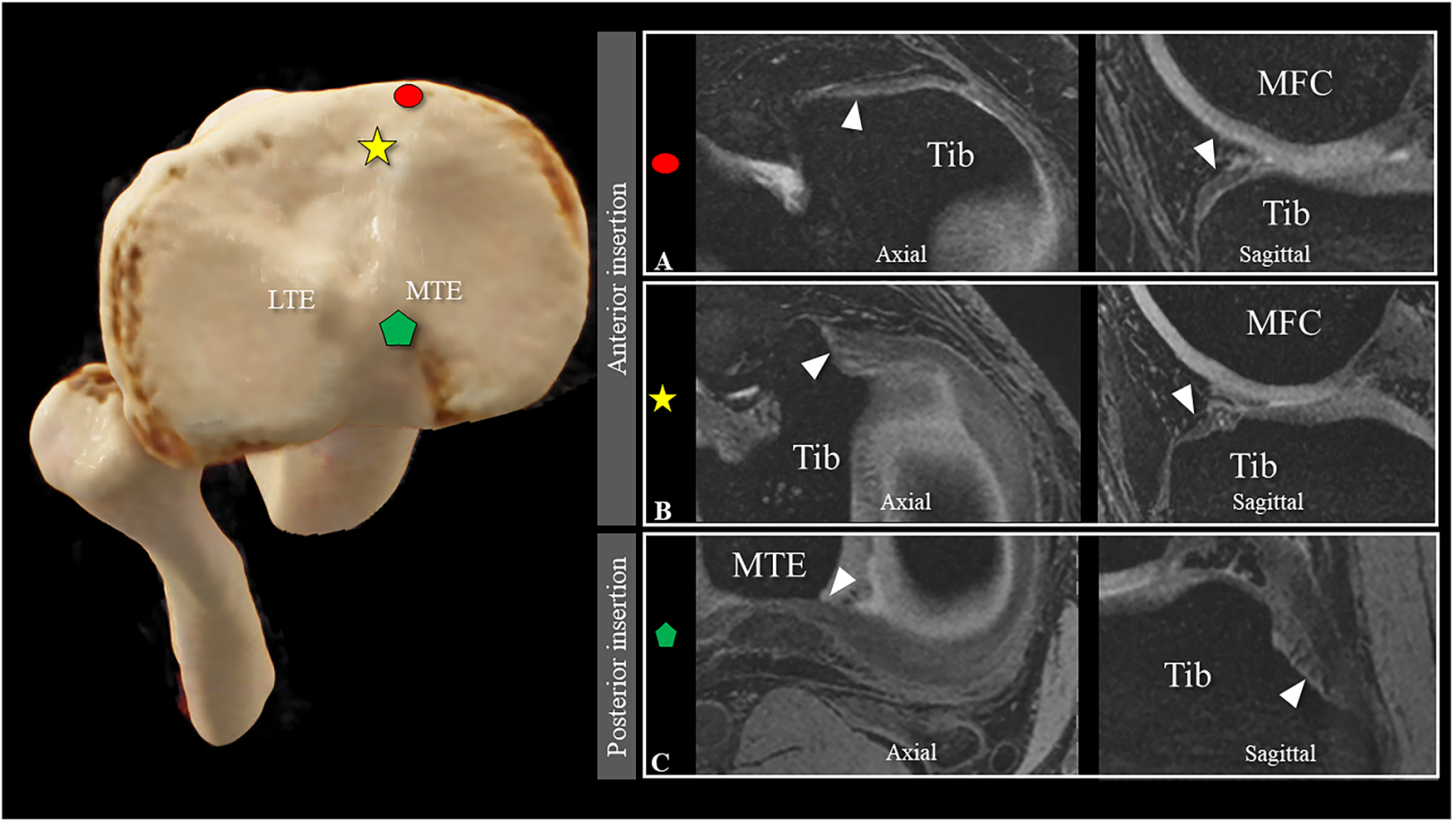
Medial meniscus insertions: The 3D DESS images in the axial and sagittal planes illustrate the two distinct insertion sites for the single root region of the anterior medial meniscus along the anterior tibia edge (Type I) (**A**) and into the intercondylar area (Type II) (**B**), respectively. The singular insertion site for a single posterior medial root into the downslope of the posterior intercondylar fossa is shown in (Type I) (**C**). Image sets **A**–**C** are all from different individuals. On the left, the different insertion sites are marked with the respective symbols on a volume-rendering depiction of a CT dataset of the proximal tibia and fibula seen from above. Tib, tibia; MFC, medial femoral condyle; MTE, medial tibial eminence; LTE, lateral tibial eminence

Both readers identified a single discoid medial meniscus (1.8% of all medial menisci; agreement with Cohen’s kappa coefficient of κ = 1). The anterior and posterior medial meniscus each exhibited a single root. The majority of the anterior medial roots (Type I) were inserted along the medial tibial edge (Fig. 3A) (71.9% (41/57)), while the remaining medial anterior roots (Type II) were inserted into the intercondylar area, anteromedial to the nearest edge and center of the ACL (Fig. 3B) (28.1% (16/57)). All posterior medial roots uniformly inserted into the downslope of the posterior intercondylar fossa (Type I) (Fig. 3C). The inter-rater reliability for assessing the number of roots and root insertion sites was perfect (κ = 1).

### Lateral meniscal variations

**Fig. 4:**
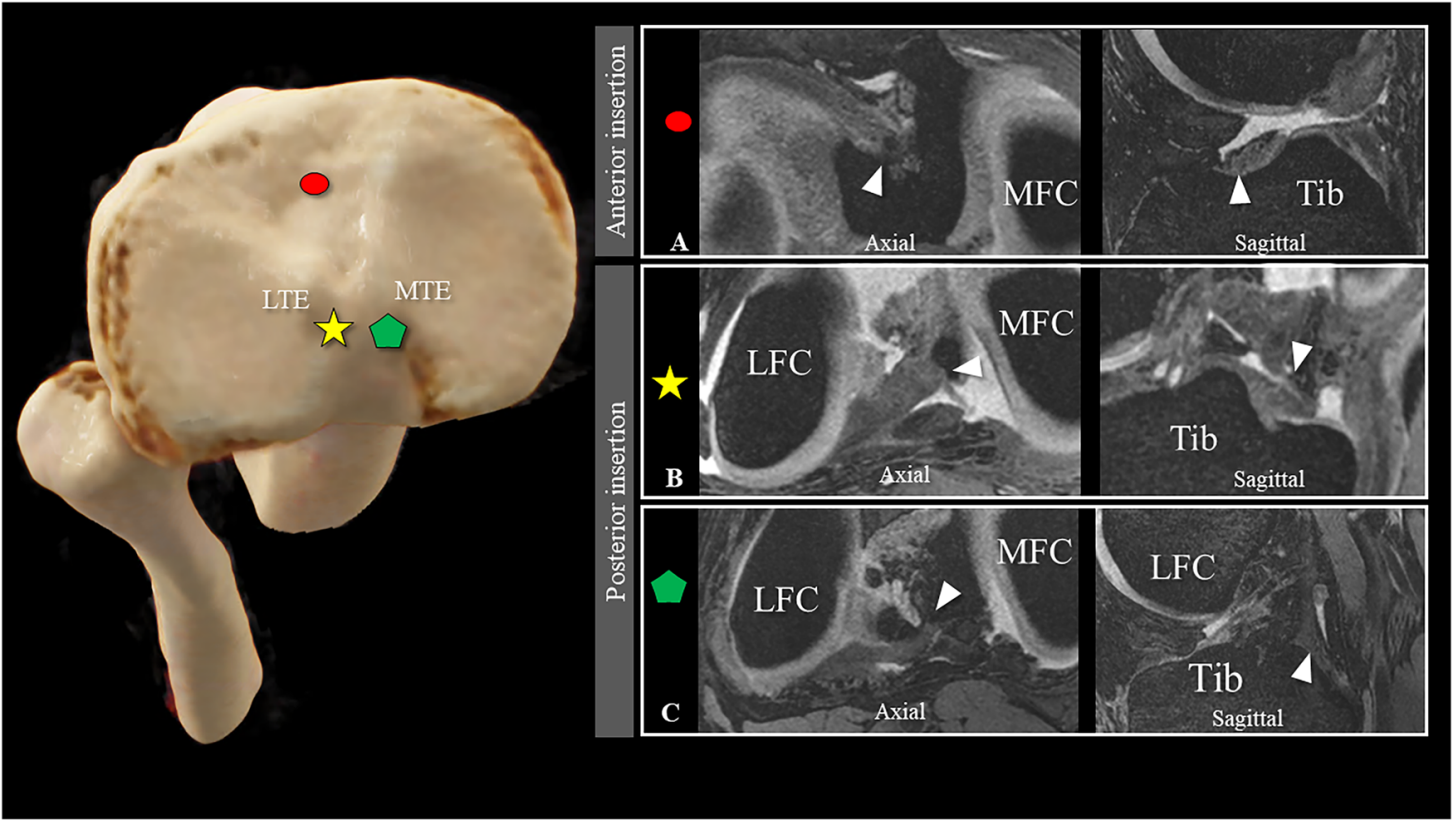
Lateral meniscus—single insertions: 3D DESS acquisition in the axial and sagittal planes illustrates the singular insertion site of the anterior lateral meniscus single root into the intercondylar area, closely adjacent to and partially overlapping the tibial insertion of the anterior cruciate ligament (ACL) (Type I) (**A**). Single insertion of the posterior lateral meniscal anchors either into the intercondylar area (Type I) (**B**) or the posterior slope of the medial eminence (Type II) (**C**). Image sets **A**–**C** are all from different individuals. On the left, the different insertion sites are marked with the respective symbols on a volume-rendering depiction of a CT dataset of the proximal tibia and fibula seen from above. Tib, Tibia; MFC, medial femoral condyle; LFC, lateral femoral condyle; MTE, medial tibial eminence; LTE, lateral tibial eminence

**Fig. 5:**
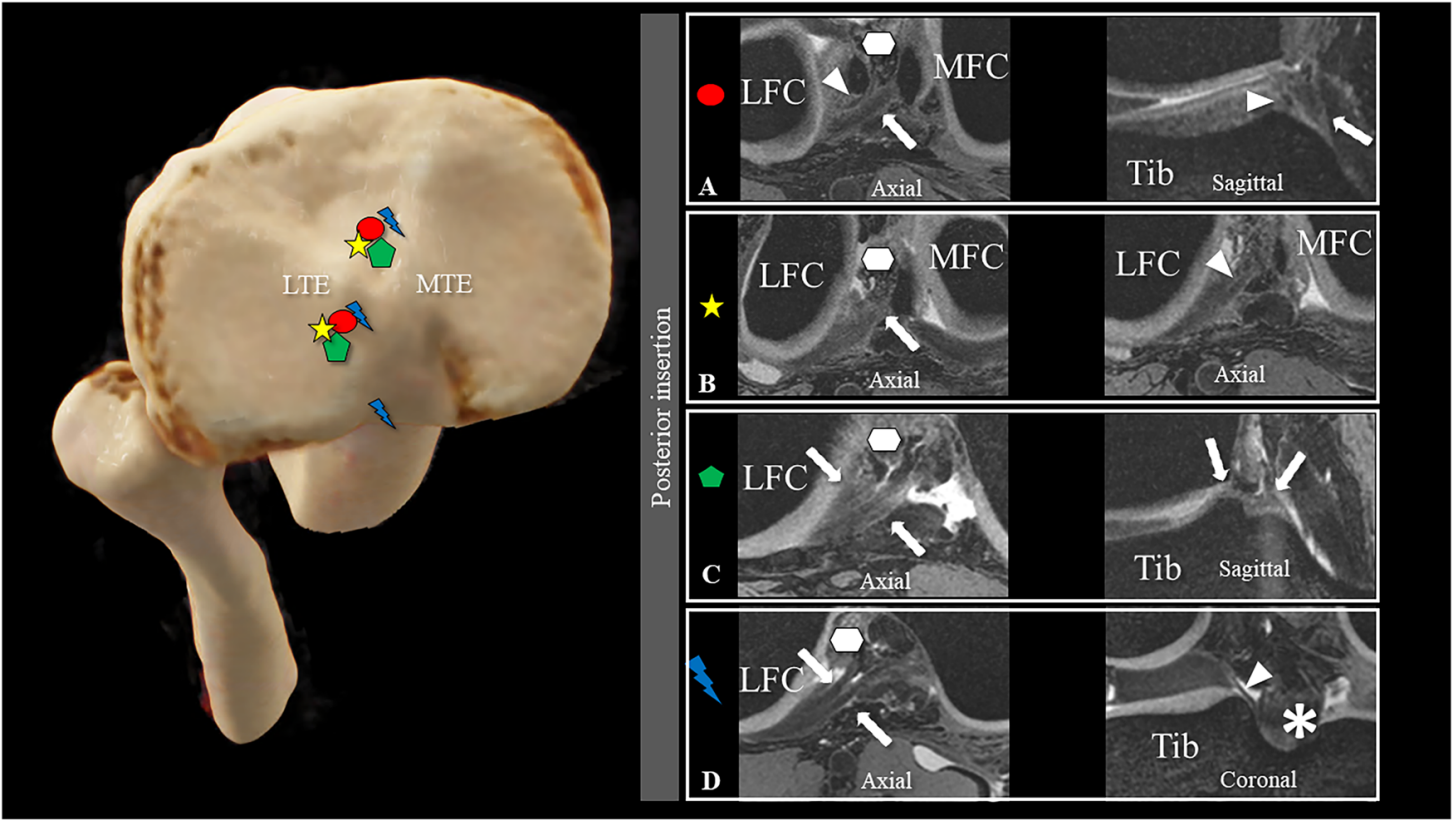
Lateral meniscus—multiple posterior insertions: 3D DESS acquisition illustrates the variations in double root insertion (**A**–**C**) and triple root insertion (**D**) across different planes: axial and sagittal (**A**, **C**), axial (**B**), and axial and coronal (**D**). **A** In the majority of double-root insertions, a major root (arrow) inserts posteromedially in close proximity to the tibial anterior cruciate ligament insertion (hexagon), whereas a minor root (arrowhead) inserts into the lateral tibial eminence (Type I). **B** In the inverted variant, the minor root inserts posteromedially near the tibial anterior cruciate ligament insertion, and the major root anchored into the lateral tibial eminence, as presented in the two axial plane images (Type II). **C** In another configuration, two equally sized root insertions anchored in the posterolateral slope of the medial and lateral eminence (Type III). **D** Triple root variant with insertions into the posterolateral slopes of the medial and lateral tibial eminences (arrow) and posterior to the tibial insertion (arrowhead) of the posterior cruciate ligament (asterisk). Image sets **A**–**C** are all from different individuals. On the left, the different insertion sites are marked with the respective symbols on a volume-rendering depiction of a CT dataset of the proximal tibia and fibula seen from above. Tib, tibia; MFC, medial femoral condyle; MTE, medial tibial eminence; LTE, lateral tibial eminence. * Posterior cruciate ligament

Both radiologists identified three lateral discoid menisci (5.3%, κ = 1), with one individual exhibiting bilateral involvement. A single anterior lateral meniscal root inserted into the intercondylar area (Type I), closely adjacent to and partially overlapping the tibial insertion of the ACL (Fig. 4A), and was consistently characterized by both readers (κ = 1). For the posterior lateral meniscus, two roots were most frequently observed (66.7% (38/57) (Fig. 5A–C)), followed by a single lateral meniscal root at 31.6% (18/57), and a rare triple lateral meniscal root at 1.8% (1/57) (Fig. 5D). Inter-rater reliability for describing the number of posterior lateral meniscal root insertions was perfect (κ = 1), and the identification of all insertion sites demonstrated strong agreement (κ = 0.808).

**Fig. 6:**
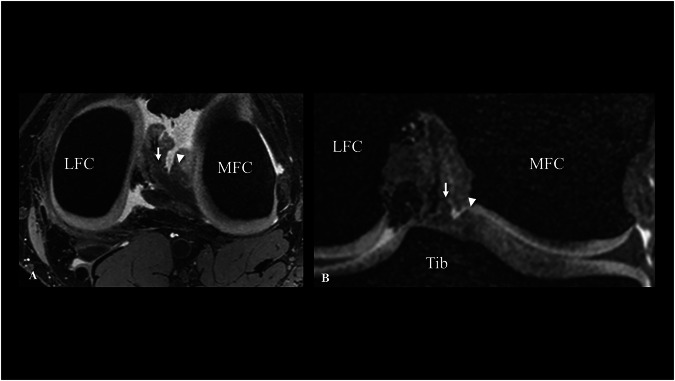
3D DESS image with double-root insertion of the posterior root of the lateral meniscus (left knee, 32-year-old female subject). The arrows highlight the major root, whereas the arrowheads indicate the minor root of the posterior lateral meniscus in the axial (**A**) and coronal (**B**) planes. Notably, in the coronal plane (**B**), a distinct double peak sign was observed, which is a characteristic associated with double root insertions (arrow and arrowhead). Tib, tibia; LFC, lateral femoral condyle; MFC, medial femoral condyle

For single posterior lateral roots, the majority (Type I) inserted into the intercondylar area (77.8% (14/18)) (Fig. 4B), while the remaining (Type II) 22.2% (4/18) anchored to the posterior slope of the medial eminence (Fig. 4C). Double root insertions predominantly inserted with a major root along the intercondylar area to the posteromedial insertion of the ACL and a minor root along the posterior slope of the lateral eminence (Type I) (Fig. 5A) (78.9% (30/38)). The inverted variant (Type II), where the major root inserts into the posterior slope of the lateral eminence and the minor root inserts next to the tibial ACL insertion, was reported in 5.3% (2/38) (Fig. 5B). The remaining double roots (Type III) inserted into the posterior lateral slopes of the medial and lateral eminence (Fig. 5C). A distinct visual feature for identifying double-root insertions was observed in 81.6% (31/38) of subjects with a double-root insertion of the posterior lateral meniscus, which manifested as a double-peak sign in the coronal plane, illustrating distinct peaks corresponding to each root before insertion (Fig. 6).

### Meniscofemoral ligaments

The prevalence of the posterior meniscofemoral (Wrisberg) ligament was higher (29/57) than that of the anterior meniscofemoral (Humphrey) ligament (20/57), accounting for 50.9% and 35.1%, respectively. Seven subjects (12.3%) exhibited no meniscofemoral ligaments, whereas one subject manifested both the Wrisberg and Humphrey ligaments simultaneously. The inter-rater reliability was strong (κ = 0.853).

Among subjects with double posterior lateral meniscal root insertions, the distribution of Wrisberg and Humphrey ligaments was balanced, with seven subjects having Humphrey ligaments and six having Wrisberg ligaments. A minority displayed either none or both ligaments, with three subjects lacking any and one possessing both ligaments. Quantitatively, the extent of the meniscofemoral ligament attachment into the posterior aspect of the lateral meniscus was 8.3 ± 1.2 mm.

### Quantitative evaluation of root insertion area and key arthroscopic landmarks

Insertion area of single meniscal root insertions ranged from 33.8 ± 4.5 mm^2^ for the posterior lateral meniscal root (inserting into the intercondylar area) to 50.2 ± 9.6 mm^2^ for the anterior lateral meniscal root (inserting into the intercondylar area) (Table 3). Total insertion area for double posterior lateral meniscal root insertions ranged from 39.3 ± 7.3 mm^2^ (roots inserting into the medial and lateral tibial eminence) to 44.7 ± 8.8 mm^2^ (major root inserting to the tibial ACL insertion and minor root inserting into the posterior slope of the lateral tibial eminence). Total insertion area of the triple posterior lateral meniscal root was 61.1 mm^2^. The quantitative relationship of all four meniscal roots to key arthroscopic landmarks is summarized in Table 4.

**Table 4:** Results of the meniscal insertion in relation to arthroscopic landmarks

	Distance (mm)
Anterior medial root insertion center to	
Medial tibial eminence apex	22.2 ± 4.3
Center of tibial tuberosity	17.7 ± 4.7
Nearest edge of ACL	9.1 ± 2.4
ACL center	16.7 ± 3
Posterior medial root insertion center to	
Medial tibial eminence apex	9.1 ± 1.7
Nearest PCL edge	3.3 ± 1.8
PCL center	6.9 ± 1.3
Anterior lateral root insertion center to	
Lateral tibial eminence apex	10.8 ± 2.1
Nearest ACL edge	3.9 ± 1.3
Center of the ACL	8.3 ± 1.7
Posterior lateral root insertion center to	
Lateral tibial eminence apex	8.8 ± 1.3
Nearest PCL edge	4.4 ± 1.4
Center of the PCL	8.3 ± 1.4

*ACL* anterior cruciate ligament, *PCL* posterior cruciate ligament

## Discussion

In this study, we demonstrated the potential of high-resolution 7-T MRI to visualize detailed meniscal anatomy. The 0.20 × 0.20 × 0.24 mm^3^ high-resolution 3D isotropic DESS sequence clearly depicted the small meniscal root insertions in exceptional detail, aiding the assessment of the posterior lateral meniscus, where most variations were observed. Notably, this includes the first description of a triple root insertion variation in the posterior lateral meniscus.

The 3D DESS sequence has been extensively used in cartilage imaging [23–25]. When combined with the increased SNR and resolution of the 7-T MRI, resulting from the higher magnetic field, its superiority in detecting chondrocalcinosis in the knee cartilage or assessing cervical nerve rootlets in microanatomical dimensions has been demonstrated [15, 26–28].

We used the 3D DESS technique to assess the fibrocartilaginous tissue of the menisci: the circumferential collagen fiber bundles of the menisci might be reflected by the fine striations observed in our study [29, 30]. Furthermore, the striated appearance of the meniscal root might align well with the histopathological structure of a dense fiber core surrounded by more loosely organized fibers [31, 32].

Previous MR imaging studies employing a 0.6 mm isotropic 3D proton density-weighted turbo spin echo (TSE) sequence revealed variations in meniscal root insertions. Ren et al conducted a retrospective study with 62 subjects using a 3-T MRI, and reported only a single insertion for each root [33]. Regarding the anterior medial root, the insertions in the intercondylar area aligned with our results. However, they noted insertions along the transverse ligament, in contrast to our observations along the medial tibial edge (Type I). Additionally, they observed a combined insertion site of the anterior medial root in the intercondylar area and the transverse ligament. However, this finding contradicts the results by Brody et al, who correlated gross anatomical findings with 3-T MRI [30]. In their study, the transverse ligament, which connects the anterior horns of the medial and lateral menisci, was identified as a distinct structure, with the medial meniscal root inserting anterior to it and the lateral root posterior to it on MRI. Therefore, we hypothesize that the observed insertion in the study by Ren et al likely represents a bony attachment in close proximity to the transverse ligament, which may have been misinterpreted as an intraligamentous insertion. To our knowledge, no arthroscopic study has been conducted to directly assess this specific anatomical relationship.

For the posterior insertion of the lateral meniscus, the majority of observed insertions were to the medial and lateral tibial eminences, which corresponds to Type III in our classification and was the least common type observed in our study. Similar to our observations, they reported three distinct insertion points (medial and lateral tibial eminence and the PCL), although in their study setup at 3 T, they did not investigate the number of root insertions.

Conversely, Wang et al prospectively evaluated 60 knees on 3-T MRI using a 3D proton density-weighted sequence and noted that for the posterior lateral meniscus root, the majority had a single root inserted into the medial eminence, while a minority exhibited double root insertions with a major and minor root, which aligned with our observations [11]. However, our study extended their results by adding three more patterns for the posterior lateral meniscus, especially the inverted double-root insertion (Type II), equal-sized root insertion (Type III), and triple-root insertion (Type I).

In a study by You et al, 103 patients with an intact posterior root of the lateral meniscus underwent 2D spin-echo MRI at 3 T using fat-suppressed proton density-weighted images with 2.5–3 mm image slice thickness [10]. The study identified that most roots inserted into two sites: the intercondylar area and the posterior slope of the lateral tibial eminence. A minority inserted solely into one site, either the intercondylar area or the posterior slope of the lateral eminence. Although the study was performed using thicker slices, the results aligned well with our observations.

In a systematic review by Deckey et al, the presence of either a Wrisberg or Humphrey ligament was 70.8%, and in 17.6% both ligaments were present [34]. Two meta-analyses by Pekala et al showed a prevalence of 70.4% and 55.5% for the Wrisberg and Humphrey ligaments, respectively [35, 36]. In our study population, the Wrisberg ligament was seen less frequently (50.9%), and the Humphrey ligament was seen more frequently (35.1%), possibly because of the different sizes and composition of the study population.

Meniscal root insertion areas vary widely across cadaveric studies. For the anterior medial root, reported values range from 47.2 ± 14.1 mm² (Pangaud et al) to 139 ± 43 mm² (Kohn et al), with intermediate measurements (56.3–61.4 mm²) (LaPrade et al, Ellman et al, Johnson et al). Our smaller area (42.7–46.3 mm²) likely reflects MRI’s selective visualization of the central attachment, which constitutes ~51% of the total area, including supplemental fibers (LaPrade et al) [9, 19, 37–39]. For the anterior lateral root, values range from 44.5 mm² (Johnson et al) to 140.7 ± 30 mm² (LaPrade et al), with intermediate values (47.4–99.5 mm²) (Pangaud et al, Oshima et al, Ellman et al and Kohn et al) [40]. Our measurement (50.2 ± 9.6 mm²) aligns with the lower range, likely due to MRI’s inability to detect fibers extending beneath the ACL (LaPrade et al). For the posterior medial root, our area (34.5 ± 6.8 mm²) closely matches 30.4 ± 2.9 mm² (Johannsen et al) but is smaller than 41.6–80.0 mm² (Ellman et al, Pangaud et al, Johnson et al, and Kohn et al), reflecting MRI’s focus on central fibers. For the posterior lateral root, our single and double insertion areas match 39.2 ± 2.4 mm² (Johannsen et al) but are lower than 48.4–115 mm² (Pangaud et al, Ellman et al, and Kohn et al) and higher than 28.5 mm² (Johnson et al). MRI likely excludes lateral meniscal attachments (Johannsen et al). Arthroscopic landmark distances showed partial alignment with prior studies. Our anterior medial root-to-ACL measurement fell between 15.9 ± 3.4 mm (Pangaud et al) and 18.2 ± 2.9 mm (LaPrade et al), while our anterior lateral root-to-ACL (5 ± 1.8 mm) was closer to 5.0 ± 1.8 mm (LaPrade et al) than 9.8 ± 2.9 mm (Pangaud et al). Posterior root-to-PCL distances were smaller than reported ranges: 11.5–12.7 mm (lateral) and 8.2–10.9 mm (medial) (Pangaud et al and Johannsen et al). For tibial eminence distances, our posterior lateral root-to-lateral tibial eminence apex exceeded 5.3 ± 0.3 mm (Johannsen et al), while other root-to-eminence distances were smaller than reported values: 11.5 ± 0.9 mm (posterior medial), 27.5 ± 3.3 mm (anterior medial), and 14.4 ± 2.2 mm (anterior lateral) (Johannsen et al and LaPrade et al). These variations likely reflect methodological differences, underscoring the limitations of imaging compared to cadaveric studies.

Previous anatomical studies have demonstrated that the meniscal root attachments are reinforced by complemental fibers, such as the shiny white fibers associated with the posterior medial root, enhancing failure strength [20, 39]. Shiny white fibers extend from the posterior medial root insertion to the tibial attachment of the anterior lateral bundle of the PCL [39]. Similarly, the supporting fibers for the posterior lateral meniscal root extend from the root insertion to the lateral side of the medial tibial eminence [20, 39, 41]. In the current study, we did not identify the supplemental fibers for the posterior medial meniscal root as a distinct structure. This may be attributed to the imaging sequence utilized, which may obscure the fine structure within the surrounding adipose tissue. A similar observation was made regarding the complemental fibers of the posterior lateral meniscal root, where it is possible that the structure was concealed by the root fascicles.

In addition, our findings align with those previously described by Johnson et al in their cadaveric study, who noted the close anatomical proximity of the medial and lateral posterior root insertions. This proximity is crucial to consider during graft placement to avoid injuring the adjacent root [9].

The MRI diagnosis of a zip lesion or Wrisberg rip, a longitudinal vertical/oblique meniscal tear, remains challenging, with a reported positive predictive value of 13% [42]. In their review by Taneja et al defined the zip lesion as a tear extending more than 14 mm in the mediolateral direction from the lateral edge of the PCL [43]. In the study by Park et al, which correlated MRI findings with arthroscopic results, a distinct structure located more than 12 mm lateral to the PCL was associated with a zip lesion, showing a sensitivity of 80% and specificity of 82% [44]. In our study, we quantified the extent of the meniscofemoral ligament attachment into the posterior aspect of the lateral meniscus. To our knowledge, this evaluation has not been performed previously. The thresholds for defining tears in this context need to be evaluated in future studies.

We acknowledge the following limitations of our study: Since only healthy participants were included, no arthroscopic correlation was available. However, by including only healthy individuals, we ensured that only the normal meniscal anatomy was analyzed.

In conclusion, anatomical variability exists predominantly for the posterior root of the lateral meniscus regarding the number of fascicles and osseous insertion points. 7 T 3D DESS imaging provides high-resolution visualization of meniscal roots and yields arthroscopic landmark measurements comparable to those reported in cadaveric studies.

## Abbreviations

ACL: Anterior cruciate ligament
DESS: Double echo steady state
MRI: Magnetic resonance imaging
PCL: Posterior cruciate ligament
SNR: Signal-to-noise ratio
T: Tesla
